# Detecting significant genotype–phenotype association rules in bipolar disorder: market research meets complex genetics

**DOI:** 10.1186/s40345-018-0132-x

**Published:** 2018-11-11

**Authors:** René Breuer, Manuel Mattheisen, Josef Frank, Bertram Krumm, Jens Treutlein, Layla Kassem, Jana Strohmaier, Stefan Herms, Thomas W. Mühleisen, Franziska Degenhardt, Sven Cichon, Markus M. Nöthen, George Karypis, John Kelsoe, Tiffany Greenwood, Caroline Nievergelt, Paul Shilling, Tatyana Shekhtman, Howard Edenberg, David Craig, Szabolcs Szelinger, John Nurnberger, Elliot Gershon, Ney Alliey-Rodriguez, Peter Zandi, Fernando Goes, Nicholas Schork, Erin Smith, Daniel Koller, Peng Zhang, Judith Badner, Wade Berrettini, Cinnamon Bloss, William Byerley, William Coryell, Tatiana Foroud, Yirin Guo, Maria Hipolito, Brendan Keating, William Lawson, Chunyu Liu, Pamela Mahon, Melvin McInnis, Sarah Murray, Evaristus Nwulia, James Potash, John Rice, William Scheftner, Sebastian Zöllner, Francis J. McMahon, Marcella Rietschel, Thomas G. Schulze

**Affiliations:** 1 Department of Genetic Epidemiology in Psychiatry, Central Institute of Mental Health, Medical Faculty Mannheim/Heidelberg University, Mannheim, Germany; 2 Department of Genomics, Life & Brain Center, University of Bonn, Bonn, Germany; 3 Institute of Human Genetics, University of Bonn, Bonn, Germany; 4 Center for Integrative Sequencing, iSEQ, Department of Biomedicine, Aarhus University, Aarhus, Denmark; 5 Department for Biostatistics, Central Institute of Mental Health, Medical Faculty Mannheim/Heidelberg University, Mannheim, Germany; 6 Human Genetics Branch, Intramural Research Program, National Institute of Mental Health, National Institutes of Health, US Department of Health and Human Services, Bethesda, MD USA; 7 Institute of Neuroscience and Medicine (INM-1), Structural and Functional Organisation of the Brain, Genomic Imaging, Research Centre Juelich, Juelich, Germany; 8 Department of Biomedicine, University of Basel, Basel, Switzerland; 9 Department of Computer Science & Engineering, University of Minnesota, Minneapolis, MN USA; 10 Department of Psychiatry, University of California San Diego, San Diego, USA; 11 BGI-Shenzhen, Beishan Industrial Zone, Yantian District, Shenzhen, China; 12 Department of Biochemistry and Molecular Biology, Indiana University School of Medicine, Indianapolis, USA; 13 The Translational Genomics Research Institute, Phoenix, USA; 14 Department of Psychiatry, Indiana University School of Medicine, Indianapolis, USA; 15 Department of Psychiatry and Behavioral Neuroscience, University of Chicago, Chicago, USA; 16 Department of Mental Health, John Hopkins Bloomberg School of Public Health, Baltimore, USA; 17 Department of Psychiatry and Behavioral Sciences, John Hopkins School of Medicine, Baltimore, USA; 18 J. Craig Venter Institute, La Jolla, USA; 19 Scripps Genomic Medicine & The Scripps Translational Sciences Institute (STSI), La Jolla, USA; 20 Department of Pediatrics and Rady’s Children’s Hospital, School of Medicine, University of California San Diego, La Jolla, USA; 21 Department of Medical and Molecular Genetics, Indiana University School of Medicine, Indianapolis, USA; 22 Department of Computational Medicine and Bioinformatics, University of Michigan, Ann Arbor, USA; 23 Department of Psychiatry, University of Pennsylvania, Philadelphia, USA; 24 University of California, San Diego, La Jolla, USA; 25 Department of Psychiatry, University of California at San Francisco, San Francisco, USA; 26 University of Iowa Hospitals and Clinics, Iowa City, USA; 27 Center for Applied Genomics, Children’s Hospital of Philadelphia, Abramson Research Center, Philadelphia, USA; 28 Department of Psychiatry and Behavioral Sciences, Howard University Hospital, Washington, USA; 29 Cardiovascular Institute, University of Pennsylvania School of Medicine, Philadelphia, PA USA; 30 Institute for Translational Medicine and Therapeutics, School of Medicine, University of Pennsylvania, Philadelphia, PA USA; 31 Dell Medical School, University of Texas at Austin, Austin, USA; 32 Department of Psychiatry, University of Illinois at Chicago, Chicago, USA; 33 Department of Psychiatry, University of Michigan, Ann Arbor, USA; 34 Department of Pathology, University of California San Diego, La Jolla, USA; 35 Department of Psychiatry, Carver College of Medicine, University of Iowa School of Medicine, Iowa City, USA; 36 Department of Psychiatry, Washington University School of Medicine in St. Louis, St. Louis, USA; 37 Rush University Medical Center, Chicago, USA; 38 Department of Psychiatry and Psychotherapy, University of Göttingen, Göttingen, Germany; 39 Institute of Psychiatric Phenomics and Genomics (IPPG), Ludwig-Maximilians-University, Munich, Nußbaumstr. 7, 80336 Munich, Germany; 40 Department of Psychiatry, Psychosomatics, and Psychotherapy, University of Würzburg, Würzburg, Germany

**Keywords:** Bipolar disorder, Subphenotypes, Rule discovery, Data mining, Genotype–phenotype patterns

## Abstract

**Background:**

Disentangling the etiology of common, complex diseases is a major challenge in genetic research. For bipolar disorder (BD), several genome-wide association studies (GWAS) have been performed. Similar to other complex disorders, major breakthroughs in explaining the high heritability of BD through GWAS have remained elusive. To overcome this dilemma, genetic research into BD, has embraced a variety of strategies such as the formation of large consortia to increase sample size and sequencing approaches. Here we advocate a complementary approach making use of already existing GWAS data: a novel data mining procedure to identify yet undetected genotype–phenotype relationships. We adapted association rule mining, a data mining technique traditionally used in retail market research, to identify frequent and characteristic genotype patterns showing strong associations to phenotype clusters. We applied this strategy to three independent GWAS datasets from 2835 phenotypically characterized patients with BD. In a discovery step, 20,882 candidate association rules were extracted.

**Results:**

Two of these rules—one associated with eating disorder and the other with anxiety—remained significant in an independent dataset after robust correction for multiple testing. Both showed considerable effect sizes (odds ratio ~ 3.4 and 3.0, respectively) and support previously reported molecular biological findings.

**Conclusion:**

Our approach detected novel specific genotype–phenotype relationships in BD that were missed by standard analyses like GWAS. While we developed and applied our method within the context of BD gene discovery, it may facilitate identifying highly specific genotype–phenotype relationships in subsets of genome-wide data sets of other complex phenotype with similar epidemiological properties and challenges to gene discovery efforts.

**Electronic supplementary material:**

The online version of this article (10.1186/s40345-018-0132-x) contains supplementary material, which is available to authorized users.

## Background

It is widely accepted that the high heritability of around 80% for bipolar disorder (BD) is conferred by a polygenic component yet to be understood in its complexity (McGuffin et al. 2003; Craddock et al. 2005). Genome-wide association studies of BD have identified several genome-wide significant variants and also hinted at the existence of many more variants which fail to achieve the rigorous threshold of genome-wide significance (p < 5.0e−08) but contribute to the overall variance when considered within the context of polygenicity (Lee et al. 2012a, b; Sullivan et al. 2012; Schulze et al. 2014). However, the number of newly identified variants is far below original expectations, with limited sample sizes being one of the explanatory factors. The largest sample for a meta-analysis of GWAS of BD to date comprised nearly 64,000 participants (Sklar et al. 2011). Although this is an impressive sample size, GWAS of other phenotypes, such as adult height, have demonstrated that samples three-times this figure are required to achieve an adequate number of significant findings (Lango Allen et al. 2010). Recent successes of the Psychiatric Genomics Consortium (https://pgc.unc.edu/) in schizophrenia genetics where case–control samples have already exceeded 100,000 individuals suggest that continued enlargement of sample size will also increase the yield of genome-wide significant findings for BD. Clinical heterogeneity of the BD phenotype may also have hampered success in identifying vulnerability genes. DSM (American Psychiatric Association 2000) and ICD (World Health Organization 2011) present a list of possible symptoms, each of which must persist for a minimum period of time for the diagnosis to be assigned. Since a diagnosis of BD is based upon the presence of a minimum number of these symptoms, the diagnosis can be assigned for varying symptom constellations. Thus the nature and number of the underlying clinical symptoms, as well as the time periods over which they occur, show substantial variation between patients. Thus, the clinical presentation is diverse, and differing disease courses are observed within each diagnostic category.

**Fig. 1:**
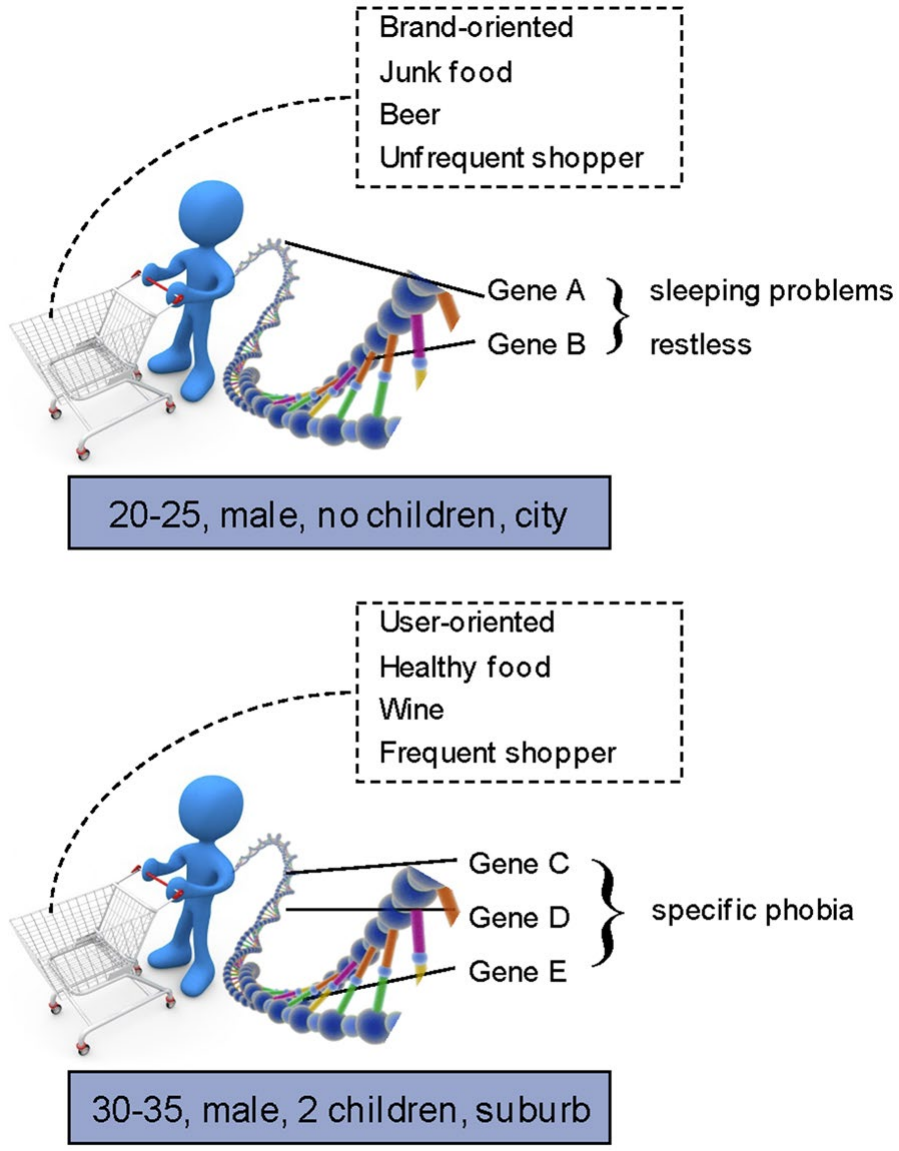
Outline of the overall approach. A main goal of market research is to identify rules that predict customer habits based on market baskets. In the cartoon, a male customer between 20 and 25 without children living in the city favours junk food and beer and when he goes shopping he will most likely buy brands. Adapting this idea to genetic research we try to identify those genetic factors from the plethora of genetic factors in the “market basket” that are characterized by specific phenotypic features (like specific phobia or restlessness). The cartoon contains graphical depictions by Benjamin Albiach Galan and Konstantinos Kokkinis

We hypothesize that heterogeneity can be reduced and the number of identified variants increased by analyzing the joint effect of several genetic variants on specific subsets of clinical items identified in BD patients (Purcell et al. 2009; Lee et al. 2011). We hypothesize that systematic data mining approaches from other fields can be applied to analyses of GWAS data. Popular methods such as support vector machines, Bayesian networks, and association rule mining (ARM) have been successfully applied in industry. ARM is one of the most important and well researched techniques of data mining (Kotsiantis and Kanellopoulos 2006). It aims to extract casual structures among sets of items in data bases for discovering and predicting regularities and has been applied extensively to market research (Agrawal et al. 1993; Ngai et al. 2009) in order to analyze customer habits. For several years now, it has been applied to biological data, in particular microarray data for gene expression analysis (Martinez et al. 2008; Liu et al. 2011). We consider this approach highly appropriate for genome-wide data, since its main goal is to unravel unknown associations between source data, i.e. customer profiles in market research, and potential targets, i.e. their buying behavior, which can then be used for target prediction (Fig. 1). Within the context of genome-wide data, the source data are genetic variants and the potential targets are symptom clusters. The aim of the present study is to apply this data mining approach to GWAS datasets of BD in order to identify yet undetected genotype–phenotype associations, searching for associations between frequently occurring genotype combinations and symptom clusters.

## Methods

### Samples

Genotype and phenotype data were obtained from three independent BD case–control samples, the US-American GAIN (1000 cases, 1033 controls) (Smith et al. 2009) and TGEN (1190 cases, 401 controls) collections (Smith et al. 2011) and the German BoMa (645 cases, 1310 controls) sample (Cichon et al. 2011). Clinical symptoms, sociodemographic and environmental features were ascertained using structured interviews (DIGS (Nurnberger et al. 1994) for GAIIN and SCID-I for BoMa (Spitzer et al. 1992). All phenotypes were retrieved from professionally curated databases (Potash et al. 2007; Fangerau et al. 2004). Detailed information on the samples can be found elsewhere (Smith et al. 2009, 2011; Baum et al. 2008). Descriptive statistics for both samples are provided in Additional file 1: Table S1. The total sample for the present study comprised n = 5579 subjects (2835 cases and 2744 controls). The GAIN sample was used for the discovery step, and the TGEN and BoMa samples were used for the replication step. Prior to study inclusion, written informed consent was obtained from all subjects.

### Selection of clinical features

In addition to the two phenotypic specifiers age at onset (AAO) and sex, we included a variety of other phenotypic feature, for the selection of which we applied the following criteria: (i) evidence of familiality and/or heritability (Schulze 2006); (ii) a frequency of at least 5% across all three samples; (iii) a missing data rate of less than 10%; (iv) availability in at least two of the three data sets; and/or (v) clinical features with a high frequency among BD patients, including co-morbid features not being part of the diagnosis of BD. In total, we selected 23 clinical features (Additional file 2: Table S2), the frequency of which was similar across all three samples (Additional file 3: Figure S1), and ranged from < 10% (e.g. eating disorder) to 80% (e.g. reckless behavior).

### Selection of single markers and genetic model

The GAIN and TGEN samples were genome-wide genotyped on the Affymetrix 6.0 SNP array. For the BoMa sample, the Illumina HumanHap550 BeadChip was used. All genotypes were imputed based on 2.1 million HapMap Phase 2 markers (McMahon et al. 2010). Due to computational runtime constraints, our analysis is based on a selected number of markers. We included only those SNPs that showed an association p-value of less than 0.001 in a recent meta-analysis of 4961 BD patients and 7294 controls (Additional file 4: Text S1, Methods-*SNP selection*). Our resulting SNP set comprised 5487 SNPs, on which LD pruning (Additional file 4: Text S1, Methods-*Linkage disequilibrium*) was performed in order to reduce redundancy within the genotype data before the discovery step and to decrease runtime. This left us with a total of 1599 SNPs. Of these, 1581 SNPs were available in all three samples studied. As the ARM approach requires binary variables we had to transform the genotype information into a binary format (Additional file 4: Text S1, Methods-*Genetic Models*).

### Algorithm for association rule mining

**Fig. 2:**
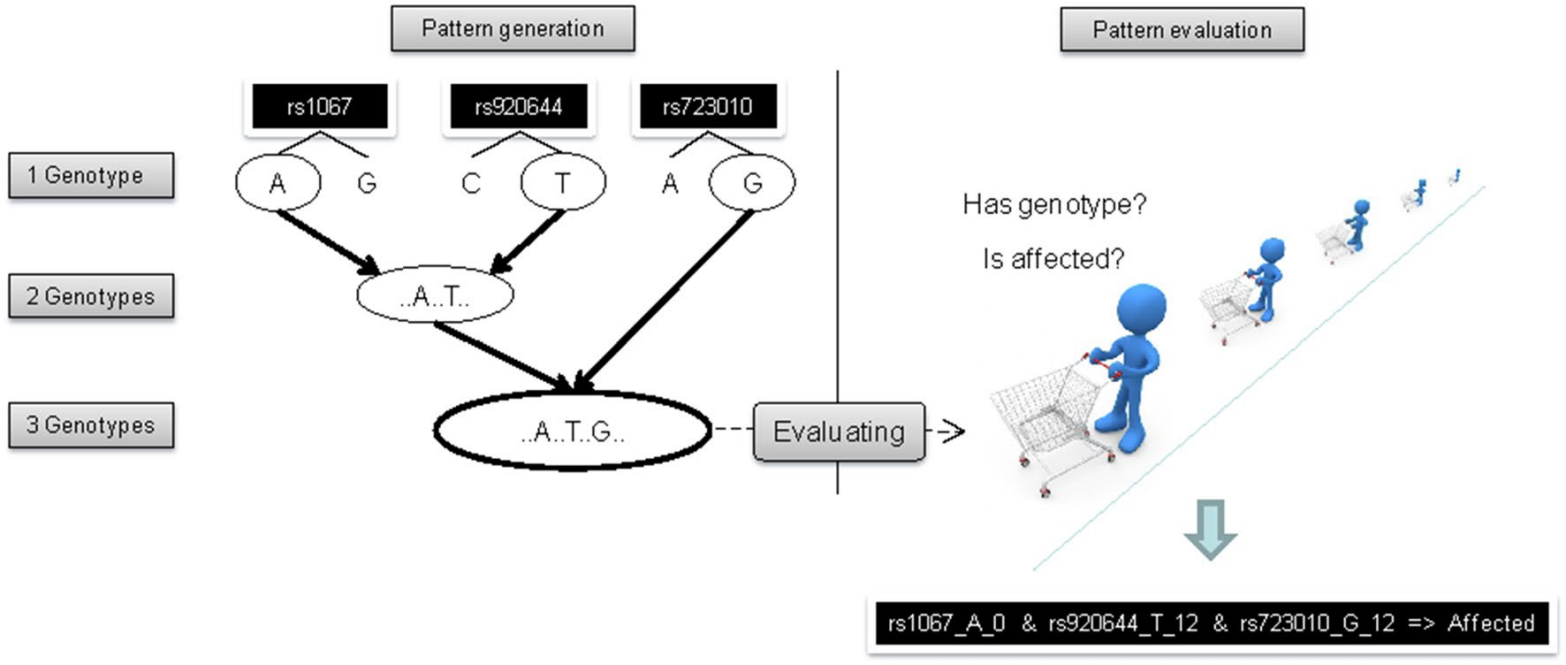
Illustration of the implemented version of the association rule mining algorithm. The lattice shown left is traversed starting from root {} to all leaves. Each genotype pattern (node in the tree) represents a subgroup of patients shown in the genotype matrix G. Additionally, using the p phenotype information of the patients from matrix P, we can count genotype and phenotype occurrences in contingency tables. Here illustrated for the genotype pattern g_1_g_2_g_n_ with ‘a’ counting all patients where genotype g_1_g_2_g_n_ and phenotype p_i_ are present, ‘d’ were neither of both are present, and ‘b’ and ‘c’ counting patients with presentation of genotype g_1_g_2_g_n_ but not phenotype p_i_ and visa versa. The lattice is traversed as long as there are unprocessed genotype patterns that cannot be pruned before

The basic idea for identifying genotype–phenotype data using these binary genotype data is to (i) receive frequent genotype patterns, (ii) to look for significantly associated phenotypes as candidates, or in terms of the original algorithm *candidate association rules*, in a discovery dataset, and (iii) to validate these candidate association rules in an independent replication dataset. Figure 2 illustrates the basic idea of combining genotypic information in order to identify frequent genotype patterns (left) and evaluate the patterns regarding interesting phenotype traits in order to receive a candidate association rule like genotype-pattern A implies phenotype-pattern B (*A *⇒ *B*) (right).

#### Identifying frequent genotype patterns

The frequent genotype patterns can be identified in a systematic manner. Several approaches have been developed for association rule mining (Han and Kamber 2006; Maimon and Rokach 2005). Here we use the most common Apriori algorithm, as it can be implemented in a straightforward manner and shows a good performance for short patterns, making it an ideal choice for the present study. For details, see Additional file 4: Text S1, Methods-*Runtime*, *Apriori algorithm*, and *Closed frequent itemsets*.

#### Discovery of candidate association rules

Once a frequent genotype pattern is identified it is tested for association with each phenotypic trait, i.e. each of the 23 selected clinical features. This step involves the generation of a contingency table for the frequent genotype pattern and each clinical feature. Based on this contingency table, the interestingness of an association rule is assessed. For details, see Additional file 4: Text S1, Methods-*Association rule discovery*.

#### Replication of candidate association rules

The third stage of our rule mining approach is the replication of the candidate rules. Significance testing is rarely investigated in rule mining (Webb 2006). However, we considered this to be important as an inherent aspect of rule mining is the occurrence of false positive results. We anticipated 50,000 false positives per one million tests on the basis of the widely used type I error rate of 5%. One approach to correct for this is to assume that the association rules are independent and apply Bonferroni correction to the test statistics in the discovery data only. However, several simulations have shown that as well as reducing the rate of false discoveries, the Bonferroni approach also reduces the rate of true positive findings (Webb 2006). Alternatively, permutation tests can be performed to test whether or not the association between a genotype pattern and a phenotype cluster is random (Additional file 4: Text S1, Methods-*Permutation tests*). However, when constrained to a single dataset, both methods are susceptible to overfitting. Thus, we considered the performance of a replication of all candidate rules n_CR_ in an independent dataset a more appropriate alternative as this adjusts for potentially spurious sample effects and random associations. Using the latter approach and the Bonferroni method, we defined a primary test-wide significance level *α*_*adj*_ for the replication as:

$$\alpha_{adj} = 0.05/n_{CR} .$$

However, as shown by our findings and those of Webb (2006), when extracting a set of association rules using the ARM approach, the rules are unlikely to be independent. Thus, this significance testing remains conservative and is likely to reject true positive results. Therefore, we also report p-values adjusted using the false discovery rate (FDR). This is an alternative statistical method to adjust for multiple testing: FDR assumes sub-groups of tests to be dependent. FDR is less conservative, resulting in an increase in power at the cost of an increased likelihood of type I errors (Benjamini and Hochberg 1995; Benjamini 2010).

### Analyses

In order to apply our approach to the GWAS data, we developed a software tool, termed RUDI (*RU*le *Di*scoverer; http://www.rudi-genetics.net; see also Additional file 4: Text S1). A rule discovery analysis of 1581 SNPs (3162 variables) and 23 phenotypic traits in the 1000 cases from the GAIN sample was performed. Around 4.286e+09 genotype patterns were tested using the following settings: (a) z-score of 5.0; (b) maximum length of the genotype pattern of 3, and (c) absolute minimum support of individuals matching the particular genotype pattern of 50 (Additional file 4: Text S1, Methods-*Parameter selection*). The runtime on an Intel Xeon X3220 with 2.4 GHz was around 18 h on a single processor using the described settings. A second run was performed to replicate the candidate rules in our replication dataset of n = 1835 BD patients (TGEN + BoMa).

## Results

N = 20,882 candidate rules satisfied the required thresholds in the discovery data set. The strongest association rule showed a p-value of 3.457e−15 (#962) and thus reached significance after correction for multiple testing using the Bonferroni method (adjusted p-value = 3.260e−04). When all candidate rules were compared, 15 disjunct phenotype clusters were observed. Of these, 11 consisted of a single clinical feature. The remaining four consisted of two clinical features (Additional file 5: Table S3).

Replication of the n = 20,882 candidate rules was then performed in our replication dataset of n = 1835 BD patients. The level of significance after adjustment using the Bonferroni method was 2.394e−06 for a default alpha of 5%. Although replication of the top finding from the discovery step (#962) failed, two rules met the significance threshold:

(i) rule #12978

rs6733011_A_0, rs4113925_T_0, rs3769745_T_0 => ‘eating disorder’ (ED)

with a p-value = 3.576e−08 and an odds ratio (OR) = 3.566 [0.95 confidence interval (CI) 2.169–5.681];

and (ii) rule #6221

rs858057_G_0, rs4757144_G_0, rs3130781_C_0 => ‘simple phobia’ (SP)

with a p-value = 1.780e−06 and an OR = 2.995 [0.95 CI 1.841–4.730]. Three further rules remained significant after FDR correction (Additional file 4: Text S1, *Further results*). A total of 1252 (6.0%) of the candidate rules reached nominal significance in the replication sample. The distribution of the p-values for all candidate rules in the replication dataset fits the expected Chi squared distribution (Additional file 6: Figure S2).

### Association finding with eating disorder

Our top finding, rule #12978, showed a genotype pattern frequency of 5.2–7.4% in the case samples and of around 5.2–5.8% in the control populations (Additional file 7: Table S4). Further details of the genotype pattern are shown in Additional file 8: Table S7. In addition to the primary replication within the discovery-replication framework, two types of permutation tests (Additional file 4: Text S1, Methods-*Permutation tests*) were performed to estimate: (a) the probability of finding a more significant association with the genotype pattern by re-sampling the phenotype; and (b) the probability of randomly choosing a genotype pattern that shows at least the same level of significance. Both reject the hypothesis of a random association based on the empirical p-values observed (4.000e−06 and 7.000e−06, respectively), based on 1e + 06 trials in the discovery data. In a subsequent step, we combined the data of all n = 2835 patients and re-evaluated rule #12978, i.e. we compared patients with and without an eating disorder (BD_ED and BD_nonED, respectively) in terms of the genotype pattern of this rule. This combined analysis of cases showed a p-value = 5.300e−14 and an OR = 4.120 [0.95 CI 2.740–6.068]. Thus, within the group of patients carrying the genotype pattern, the frequency of a co-morbid eating disorder is increased on average by a factor of 4. An association analysis was then performed for each of the three SNPs of the genotype pattern to determine whether the observed association was due to the combination of the three SNPs or conferred by only one of them. Single trend tests for the phenotype ‘eating disorder’ was performed in each of the three datasets using PLINK (Purcell et al. 2007). No significant evidence was found to support the hypothesis that the association with the phenotype of the rule is driven by a single SNP (Additional file 9: Table S6).

We furthermore performed an association study of the genotype pattern of rule #12978 in cases versus controls. No differential distribution of the genotype pattern was observed between (a) the BD_nonED cases and controls and (b) between all BD cases and controls: However, the genotype pattern was significantly associated with BD_ED cases compared to controls (p-value = 4.937e−14, OR = 4.107 [0.95 CI 2.735–6.040]) (Additional file 10: Table S5).

### Association finding with simple phobia

The second finding, rule #6221, showed an association with ‘simple phobia’. The genotype frequencies were 5.4–6.9% in cases and 5.1–7.2% in controls. In the combined analysis of all cases, we observed a p-value = 3.476e−13 (adjusted p-value = 3.427e−02) and an OR = 3.551 [0.95 CI 2.453–5.063]. Thus, within the group of patients carrying the genotype pattern, the frequency of a co-morbid simple phobia increased on average by a factor of 3.5. As was the case for the rule including eating disorder, the association was not conferred by the single SNPs taken separately but only in combination (Additional file 9: Table S6). Likewise a case–control analysis showed: (a) no differential distribution of the genotype pattern between the BD_nonSP cases and controls nor (b) between all BD cases and controls. However, we observed (c) a significant differential distribution between BD_SP cases and controls (p-value = 1.686e−11, OR = 3.195 [0.95 CI 2.220–4.523]) (Additional file 10: Table S5).

## Discussion

Application of the ARM data mining approach identified significant associations between sets of candidate SNPs and BD subgroups characterized by two specific comorbid conditions: eating disorder and simple phobia.

Our top finding (rule #12978) highlights an association between the genotype pattern of rule #12978 and the subgroup of BD patients with an eating disorder. The association was conferred by the combination of three SNPs but not by the individual SNPs. While the proportion of BD patients with an eating disorder was very small (n = 192 patients; i.e. 6.8% of our sample), this frequency is comparable to that reported in other studies (McElroy et al. 2006, 2011). Thirty-seven of these patients displayed the genotype pattern of rule #12978, which was present in 182 of all BD patients. Despite the small sample size, the association finding (p-value = 4.937e−14) is rather strong with an OR = 4.107 in the combined case–control analysis, an effect size typically not seen for diagnosis-based studies.

The likelihood that our findings may be due to chance is further decreased when considering the following two points: Firstly, the replication sample was comprised of two smaller samples, and in both of these samples, the effect was in the same direction (with test-wide significance being achieved in neither). Secondly, our findings fall in line with reports on the function of the genes involved. SNP rs3769745 of rule #12978 is located in the intron region of the cyclic nucleotide gated channel alpha 3 gene (*CNGA3*) on chromosome 2. In humans, *CNGA3* is implicated in total color blindness (achromatopsia) (Ding et al. 2010; Lam et al. 2011). Animal studies have shown that *CNGA3* is required for normal vision (Biel et al. 1999), olfactory signal transduction (Leinders-Zufall et al. 2007), and involved in nociceptive processing (Heine et al. 2011). Further, it is expressed in the mouse brain and is reported to influence synaptic plasticity and behaviour (Michalakis et al. 2011). Research has also shown that the specialized olfactory subsystem to which CNGA3 belongs is required for the acquisition of socially transmitted food preferences (STFPs) in mice. Mice that lack this gene fail to acquire STFPs from other mice, and exhibit an absence of neuronal activation of the ventral subiculum of the hippocampus, a brain region implicated in STFP retrieval (Munger et al. 2010). According to the KEGG Database, *CNGA3* is in a common pathway, i.e. olfactory transduction (KEGG ID hsa04740), with *CALM1*, a candidate gene for anorexia nervosa (Pinheiro et al. 2010). To the best of our knowledge, no association between this variant and eating disorder has been reported so far. For the other two variants, a plausible support from biological data is not available. SNP rs6733011 is located in an intron region of the KIAA1211-like (*KIAA1211L*) gene on chromosome 2 that encodes the uncharacterized protein *C2orf55* (chromosome 2 open reading frame 55). The location is within a 500 kb window to rs3769745, but not in the same LD block (r^2^ = 0.027 and D’ = 0.343 in the discovery dataset). Its function remains unknown. SNP rs4113925 is located on chromosome 12q24.21 in an intron of the T-box transcription factor (*TBX5*) gene. This T-box gene has been implicated in heart development and disease as well as specification of limb identity (Wang et al. 2011).

To investigate whether our finding identified genetic markers specific to BD with an eating disorder subphenotype or eating disorder per se, we tested a potential association of the genotype pattern of rule #12978 with an eating disorder phenotype comprising anorexia and bulimia in a population-based sample from Australia (n = 1672, 12.9% with a diagnosis of anorexia or bulimia). We did not see an association of the genotype pattern of rule #12978, suggesting that our approach has detected a genetic marker for BD with comorbid eating disorder rather than for eating disorder per se.

Our second finding, rule #6221, showed an association with simple phobia. Two of the three contributing SNPs are located within genes. SNP rs4757144 is located in an intron region of the aryl hydrocarbon receptor nuclear translocator-like (*ARNTL*) gene, and rs3130781 is located in an intron region of the diffuse panbronchiolitis critical region 1 (*DPCR1*) gene. The third SNP, rs858057, is located at an intergenic region of 20p11.21. An implication of ARNTL in the etiology disorders via its influence on the circadian system has been discussed repeatedly (Mansour et al. 2006; Le-Niculescu et al. 2009; Nakatani 2006; Nievergelt et al. 2006; Shi et al. 2008; Sipilä et al. 2010). There is further report that genes homologous to *ARNTL* may be implicated in the etiology of anxiety. Sipilä and colleagues (Sipilä et al. 2010) tested several anxiety phenotypes for association with 13 circadian genes and found association between social phobia and *ARNTL2*. Thus the *ARNTL* gene family may be involved in this co-morbid phenotype. The second gene, *DPCR1*, is located in the major histocompatibility complex (MHC), which hosts genes that are crucial for the functioning of the immune system.

While we observed several other genotype–phenotype rules that may warrant further in-depth investigation (Table 1 and Additional file 4: Text S1, *Further results*), we focused on the rules implicating BD subtypes with comorbid eating disorder and simple phobia, respectively, as only these two survived our stringent multi-tiered evaluation of potential type I error. These steps help minimize—if not eliminate- type I error rate in ARM due to the overfitting of rules in a particular dataset (Han and Kamber 2006).

**Table 1:** Top 10 association rules regarding their p-values in the replication dataset (TGEN + BoMa)

PID	Groups				Statistics		Adjusted p-value	
	GP	Gp	gP	gp	p_chisq	Odds ratio (0.95 CI)	Bonferroni	FDR
12978	25	105	107	1598	3.576e−08	3.566 [2.169–5.681]	0.00075	0.00075
6221	26	84	162	1563	1.780e−06	2.995 [1.841–4.730]	0.03717	0.01859
12681	33	103	187	1512	4.648e−06	2.596 [1.682–3.917]	0.09706	0.02771
12981	25	129	107	1574	5.720e−06	2.860 [1.751–4.520]	0.11944	0.02771
6225	25	84	163	1563	6.635e−06	2.862 [1.747–4.545]	0.13855	0.02771
6228	26	93	162	1554	1.585e−05	2.690 [1.661–4.225]	0.33102	0.05517
4428	31	88	212	1504	2.021e−05	2.505 [1.600–3.830]	0.42198	0.06028
6111	21	109	111	1594	4.096e−05	2.779 [1.636–4.529]	0.85530	0.10654
6183	20	66	168	1581	4.592e−05	2.864 [1.652–4.765]	0.95887	0.10654
6178	20	68	168	1579	7.577e−05	2.777 [1.604–4.611]	1	0.15823

Listed are the rule identifier (PID), the counts per group of the contingency tables, the p-values based on the Chi squared test along with the odds ratios including confidence intervals (CI), and results from two multiple correction methods (based on 20,882 tests). The coding of the groups is as follow: G, if genotype pattern is present, g if not; P, if phenotype pattern is present, p if not. *FDR* false discovery rate

We would like to point out that the two reported association rules were associated with very low frequency phenotypes. This is due to the characteristics of the z-score approach applied. Since small proportions of the data are more likely to deviate strongly from the random distribution, larger effects and thus larger z-scores are expected. As only those rules that show a z-score of greater or equal 5 are extracted as candidates, this particular rule measure is biased towards associations with low frequency phenotypes. This further explains the relatively small number, i.e. 4 out of 15 (Additional file 4: Text S1, Methods-*Phenotype cluster*), of phenotype clusters that consisted of more than one phenotype. We may thus have discarded many potentially true findings. Given that our study can be considered a proof-of-concept for the application of ARM on GWAS-derived data for a complex phenotype, we opted for statistical stringency rather than a merely exploratory pattern mining. While this approach resulted in only two findings, they are characterized by effect sizes up to four times larger than typically seen in GWAS of BD or other complex traits.

## Conclusions

In summary, using already available GWAS data sets on BD, we have established and implemented a novel data mining process for complex genetic data. We identified genotype–phenotype patterns highlighting subtypes of BD characterized by specific comorbid conditions. These two comorbid conditions, eating disorder and simple phobia, may delineate more homogenous subgroups of BD that warrant further study in genomic studies of BD.

An important limitation of our approach is that our approach was only based on 5487 SNPs that showed some evidence of association with BD. As association rule mining may detect hidden association of specific phenotypes with previously un-identified SNPs, our approach may have missed several novel associations. This restriction, however, was due to our motivation to perform genotype–phenotype dissection on SNPs that showed some evidence of association. We were further bound by some computational runtime constraints. Further extensions of the algorithm will be required to allow for a variety of assumed genetic models (here we used a dominant genetic model), to optimize computational feasibility for an increased number of SNPs (Additional file 4: Text S1, Methods-*Runtime*), and to determine the optimal correction methodology for highly correlated data.

Our approach highlights a strategy for genotype–phenotype dissection and for the identification of genetic susceptibility variants beyond initial GWAS of heterogeneous disorders. Finally, our results emphasize the importance of thorough phenotyping, particularly with regard to comorbidity.

## Additional files

**Additional file 1: Table S1.** Descriptive data for patients with bipolar disorder and controls.

**Additional file 2: Table S2.** Details on the 23 phenotypic traits included into the study.

**Additional file 3: Figure S1.** Frequencies of the selected phenotype variables in the patients for each bipolar disorder sample. Non-binary variables are mapped to binary variables. Abbreviations: aao = age at onset; M = during mania; D = during depression.

**Additional file 4: Text S1.** Supplementary notes on methods.

**Additional file 5: Table S3.** Overview of all distinct phenotype clusters received from the candidate rules of the discovery step.

**Additional file 6: Figure S2.** QQ-Plot of all chi-squared values of the replications step (n=20,882). Expected quantiles are based on a 1 degree of freedom distribution. Confidence intervals are based on a 5% error rate. The inflation factor was estimated to be 1.140.

**Additional file 7: Table S4.** Association results for each data set of our top finding, pattern #12978.

**Additional file 8: Table S7.** Details regarding the genotype patterns of the top 5 association rules.

**Additional file 9: Table S6.** Single SNP association results for each SNP of our two test-wide significant findings (Bonferroni) regarding the phenotype cluster of the corresponding association rule.

**Additional file 10: Table S5.** Association results of the case-control analyses for the top 5 replication patterns.
