# Supplementary material for: Detecting significant genotype–phenotype association rules in bipolar disorder: market research meets complex genetics
Source: Int J Bipolar Disord. 2018 Nov 11;6:24. doi: 10.1186/s40345-018-0132-x (PMC6230336; doi:10.1186/s40345-018-0132-x)
Supplement: Supplementary file 1 — Additional file 1: Table S1. Descriptive data for patients with bipolar disorder and controls. [file 40345_2018_132_MOESM1_ESM.doc]

**Table S1.** Descriptive data for patients with bipolar disorder and controls.

|  | Discovery |  |  | Replication |  |  |  |  |
| --- | --- | --- | --- | --- | --- | --- | --- | --- |
|  | GAIN |  |  | TGEN |  |  | BoMa |  |
|  | Patients | Controls |  | Patients | Controls |  | Patients | Controls |
| Investigated individuals | 1000 | 1033 |  | 1190 | 401 |  | 645 | 1310 |
| Genotyping method | Affymetrix Human SNP 6.0 | Affymetrix Human SNP 6.0 |  | Affymetrix Human SNP 6.0 | Affymetrix Human SNP 6.0 |  | Illumina HumanHap550 | Illumina HumanHap550 |
| Males (in %) | 499 (49.9) | 532 (51.5) |  | 406 (34.1) | 238 (59.4) |  | 312 (48.4) | 665 (50.7) |
| Bipolar disorder type I  (in %) | 1000 (100) | n.a. |  | 1113 (93.5) | n.a. |  | 643 (99.7) | n.a. |
| Bipolar disorder type II  (in %) | 0 (0) | n.a. |  | 0 (0) | n.a. |  | 1 (0.2) | n.a. |
| Schizoaffective disorder  (in %) | 0 (0) | n.a. |  | 77 (6.5) | n.a. |  | 1 (0.2) | n.a. |
| Mean age at recruitment  in years (s.d.) | 42.2 (12.9) | 52.2 (17.6) |  | 42.9 (12.5) | 52.4 (15.9) |  | 44.0 (13.0) | 50.4  (11.4) |
| Mean age of onset in  years (s.d.) | 19.3 (9.3) | n.a. |  | 18.5 (9.4) | n.a. |  | 28.1 (11.0) | n.a. |

Listed are only cases and controls with sufficient phenotype data. Abbreviations: n.a. = not applicable; s.d. = standard deviation
