# Supplementary material for: Detecting significant genotype–phenotype association rules in bipolar disorder: market research meets complex genetics
Source: Int J Bipolar Disord. 2018 Nov 11;6:24. doi: 10.1186/s40345-018-0132-x (PMC6230336; doi:10.1186/s40345-018-0132-x)
Supplement: Supplementary file 2 — Additional file 2: Table S2. Details on the 23 phenotypic traits included into the study. [file 40345_2018_132_MOESM2_ESM.docx]

**Table S2.** Details on the 23 phenotypic traits included into the study.

|  |  | GAIN & TGEN |  |  | BOMA |  |
| --- | --- | --- | --- | --- | --- | --- |
| Name of variable | Description of Variable | Variable in DIGS4 (Bipolar Phenome Database)  [interview section / question] | Coding type |  | Variable in SCID/OPCRIT (IPGS database)  [interview section / question] | Coding type |
| Migraine | Has subject ever had migraine | Medical History / B1 | I |  | - | - |
| fidgety or restless (D) | Fidgety or restless during depression | Depression / F8 | I |  | - | - |
| moving or speaking slowly (D) | Moving or speaking slowly during depression | Depression / F9 | I |  | - | - |
| feelings of guilt (D) | Guilt during depression | Depression / F12 | I |  | Major Depression / A66 | I |
| frequent death/suicide thoughts (D) | Frequent thoughts about death/suicide during depression | Depression / F15 | I |  | Major Depression / A70 | II |
| grandiose thoughts (M) | Grandiose thoughts during mania | Mania / G9 | I |  | Mania / A130 | II |
| reckless behaviour (M) | Reckless behavior during mania | Mania / G12 | I |  | Mania / A138 | II |
| auditory hallucinations | Ever had auditory hallucinations | Psychosis / K1a | I |  | Psychosis / B16 | II |
| visual hallucinations | Ever had visual hallucinations | Psychosis / K1b | I |  | Psychosis / B19 | II |
| Delusions | Ever had delusions | Psychosis / K1c | I |  | Psychosis / B1 | II |
| suicide attempt | Ever attempted suicide | Suicide / O1 | I |  | Suicide / X1 | II |
| Obsessions | Ever had obsessions | Anxiety Disorders / P1 | I |  | F85 | II |
| compulsions | Ever had compulsions | Anxiety Disorders / P2 | I |  | F89 | II |
| panic disorder | Ever had panic disorder (diagnostic level) | Best Estimate | I |  | F19 | I |
| agoraphobia | Ever had agoraphobia (diagnostic level) | Best Estimate | I |  | F41 and F23 | I |
| eating disorder | Ever had anorexia or bulimia (diagnostic level) | Best Estimate | I |  | H5 and F17 | I |
| Alcoholism | Ever had alcoholism (diagnostic level) | Best Estimate | I |  | E15 | I |
| substance abuse | Ever had substance abuse problems (diagnostic level) | Best Estimate | I |  | OP78-80 | I |
| simple phobia | Ever had simple phobias (diagnostic level) | Best Estimate | I |  | F74 | I |
| social phobia | Ever had social phobias (diagnostic level) | Best Estimate | I |  | F61 | I |
| aao < 22, aao < 19 | Age at onset of disorder (first major depression or first mania, whichever is earlier) | Best Estimate | III |  | OP4 | III |
| Sex | Sex of subject | Demographics | IV |  | Demographics | IV |

The first two columns show the name of the variable and its description. The following columns show where the data are based on. Due to distinct interviews for the particular data sets coding is listed for DIGS and SCID/OPCRIT separately. Abbreviations: I = [missing, no, yes]; III = [numeric value, missing]; II = [0=missing, 1=no, 2=yes, but do not meet all criteria, 3=yes]; IV = [missing, male, female]
