# Supplementary material for: Detecting significant genotype–phenotype association rules in bipolar disorder: market research meets complex genetics
Source: Int J Bipolar Disord. 2018 Nov 11;6:24. doi: 10.1186/s40345-018-0132-x (PMC6230336; doi:10.1186/s40345-018-0132-x)
Supplement: Supplementary file 3 — Additional file 3: Figure S1. Frequencies of the selected phenotype variables in the patients for each bipolar disorder sample. Non-binary variables are mapped to binary variables. Abbreviations: aao = age at onset; M = during mania; D = during depression. [file 40345_2018_132_MOESM3_ESM.docx]

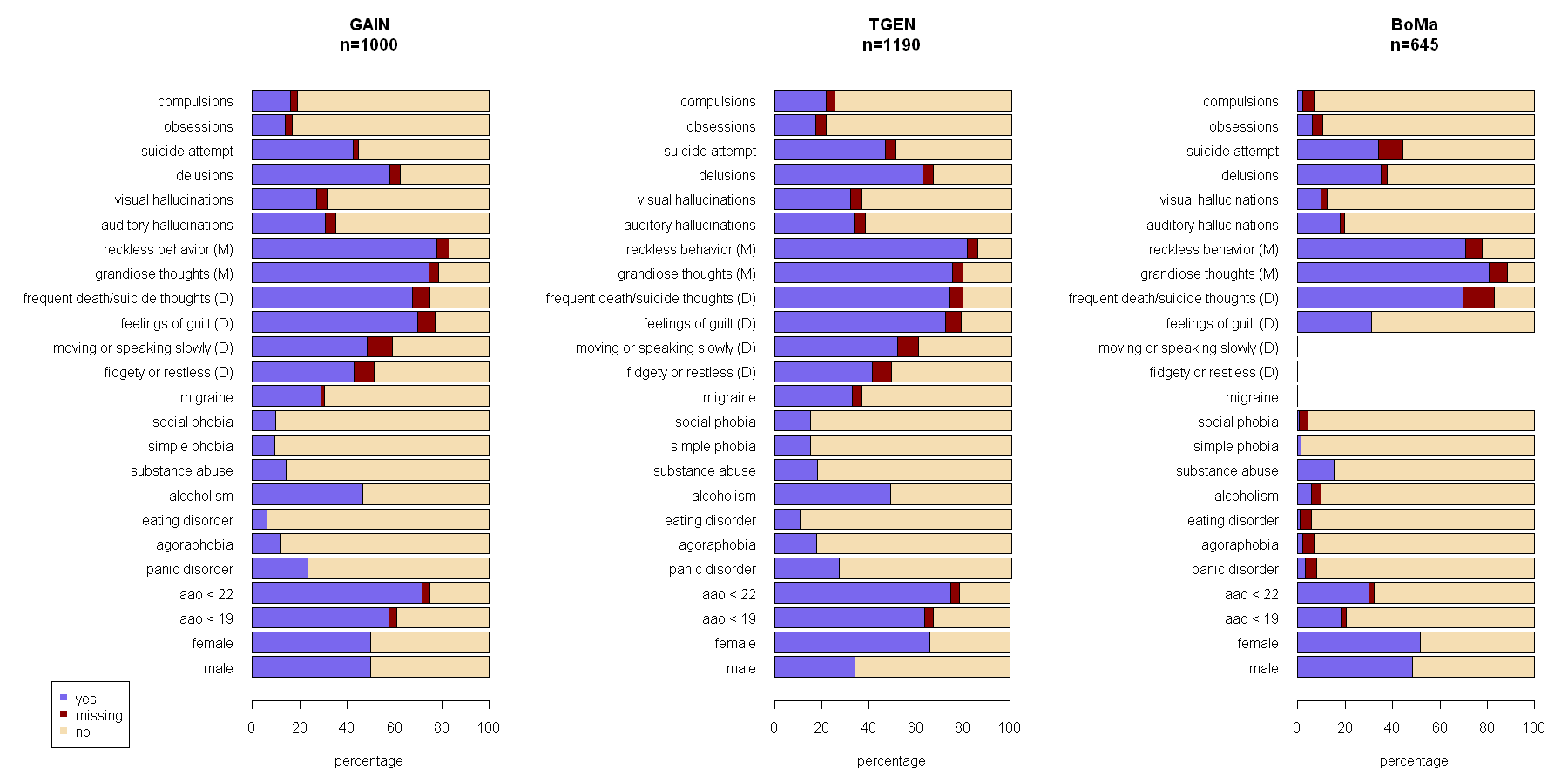


**Figure S1.** **Frequencies of the selected phenotype variables in the patients for each bipolar disorder sample.** Non-binary variables are mapped to binary variables. Abbreviations: aao = age at onset; M = during mania; D = during depression.
