# Supplementary material for: Detecting significant genotype–phenotype association rules in bipolar disorder: market research meets complex genetics
Source: Int J Bipolar Disord. 2018 Nov 11;6:24. doi: 10.1186/s40345-018-0132-x (PMC6230336; doi:10.1186/s40345-018-0132-x)
Supplement: Supplementary file 4 — Additional file 4: Text S1. Supplementary notes on methods. [file 40345_2018_132_MOESM4_ESM.docx]

Additional file

Detecting significant genotype-phenotype association rules in bipolar disorder: market research meets complex genetics

**METHODS**

**SNP selection.** For the selection of SNPs a subset of individuals of the replication dataset has been used, i.e. the cases of the BoMa sample. As they account for a negligible amount (5.3%) of the individuals used in the meta-analysis that comprised 12,255 individuals a significant bias on the results of the replication step is unlikely.

**Linkage disequilibrium (LD).** Linkage disequilibrium describes a non-random association of alleles/genotypes at two or more loci. While several measurements can potentially be used to describe this correlation, two of them are commonly used in genetic research: D’ and r² [1]. If multiple loci within a certain range show high LD between each other the region is referred to as a region of strong LD. These regions are of interest from a biological point of view, e.g. in the estimation of the allele age [1]. However, from an analytical point of view they hold redundant information. While the number of independent tests (and as a consequence the number of independent detectable association rules) stays the same, the number of overall tested association rules (and the necessary computation time) increases with the degree of LD in the data used for the analysis. A commonly used technique to reduce the amount of redundant information (so called LD-pruning) drops SNP marker from analysis that show a high LD with a SNP in its direct neighborhood. For this paper, we used a sliding window approach as implemented in PLINK [2] that drops a SNP if in a certain neighborhood (n=20 consecutive SNPs) a SNP with a LD above a defined threshold (r²=0.8) exists. After the LD is calculated for all pairwise combinations within the window of 20 consecutive SNPs the window is moved forward using a 3 SNP step width. We applied this method with PLINK’s option ‘--indep-pairwise 20 3 0.8’ and received n=1,581 SNPs that were available in all three samples. The applied threshold (r²=0.8) is rather conservative and removes only SNPs showing strong LD.

**Genetic models.** Genetic research in humans usually is based on single genetic loci with two alleles, particularly two different bases. This is particularly important for analysis purposes. Let D be the minor allele, d be the major allele, and DD, Dd, and dd all possible genotypes for a single locus, than D vs. d is the allelic model, (DD,Dd) vs. dd the dominant model, DD vs. (Dd,dd) the recessive model, and DD vs. Dd vs. dd the genotypic model. In general, the allelic model has more power to detect a true finding if the true underlying model is unknown. One reason is that it is based on 2 groups only which produces less groups with low counts (e.g. < 5) compared to the genotypic model. But the allelic model has a greater bias if the frequencies are non-random distributed [3]. Due to the expectation of low frequent genotypes, we use the dominant model on the minor allele as a trade-off between power and bias by non-random distribution. Here, we use SNP rs6733011 to illustrate the process of collapsing the genotype information Rs6733011 has the three genotypes GG, GA, and AA, where G and A are the major and minor alleles, respectively. The related genotype variable is mapped to two binary variables $v_{1}=rs6733011\_A\_12=\left\{\begin{matrix} 1, if GA\wedge AA \\ 0,else \end{matrix}\right.$, and $v_{2}=rs6733011\_A\_0=\left\{\begin{matrix} 1, if GG \\ 0,else \end{matrix}\right.$. $v_{1}$and$v_{2}$are complementary and carry identical information. This procedure, however, is required as ARM only checks if a genotype is present or not. By applying this mapping to all 1,581 genotype variables, we obtained a set of 3,162 binary variables. For reasons of computational and interpretative simplicity, we chose a dominant model of analysis for this first adaptation of ARM to genotype-phenotype analysis in complex genetics. Other models and extensions to larger number of markers and phenotypes are subject of further research and development.

**Runtime.** In association rule mining algorithms, runtime exponentially increases with a linearly increasing number of variables. Formally, the number of possible combinations of patterns is $2^{\left|I\right|}$ ($I$= set of variables), when no constraints are introduced and when variables are binary. When several pruning techniques are applied, not all of the potential combinations need to be investigated. A parameter that has a major impact on the runtime is the length of the genotype pattern. By setting an upper bound, the total number of expected combinations is reduced to $\left(\begin{matrix} n \\ k \end{matrix}\right)$, the binomial coefficient[4], which represents a substantial decrease. Regarding the setting in this paper the number of investigated genotype patterns is reduced from $2^{\left|I\right|}=2^{3162}$ to $\left(\begin{matrix} \left|I\right| \\ k \end{matrix}\right)=\frac{\left|I\right|!}{(\left|I\right|-k)!k!}=\frac{3162!}{3159!\times3!}=5.264e09\approx2^{33}$. The binomial coefficient here is only an upper bound as the implicit pruning techniques and the minimum support parameter further influence the exact number of investigated patterns. As the association between the runtime and the minimum support is logarithmic and asymptotically approaches the maximum runtime at the lowest minimum support threshold, i.e. 1, this parameter has only a small impact compared to the previous ones. In total the pruning techniques and the *min_sup* threshold further reduced the number of investigated genotype patterns to 4.2863E+09, a further reduction by nearly 20%. The relationship between sample size and runtime is linear. Increasing the sample size by a factor of 10 will lead to an increase in runtime by the same factor only. Thus, the algorithm is applicable to large sample sizes as used within the recent consortia. The threshold of the z-score does not have an impact on the runtime. In summary, the allowed length of the genotype pattern and the number of available genotype variables are the major parameters to regulate the runtime of a certain analysis. Both need to be constrained in a way to achieve feasible runtimes. The remaining parameters are rather negligible, even if a slight improvement in runtime can be achieved by increasing the minimum support.

**Apriori algorithm.** The Apriori algorithm takes advantage of the monotonicity of frequencies. Particularly, it allows for an efficient filtering of frequent subpatterns of any parent pattern by assuming that the subpattern cannot occur more frequently than the parent pattern. Let A and B be two events. A occurs 3 times and B occurs 2 times. The monotonicity of frequencies now states that both events, A and B, can only occur 2 times at once. Thus, by applying a frequency filter of n=3, a pattern B and all subpatterns containing B can be filtered out and the number of patterns to be investigated can be reduced substantially.

**Closed frequent itemsets.** A closed frequent itemset is an itemset that does not have an immediate superset with the same support as the itemset itself. E.g. let *X*={a,b,c} be an 3-itemset with support(*X*)=10. If there is no superset *X*_s_={a,b,c,y} with support(*X*_s_)=10 than *X* is a closed itemset. Using this property, a more efficient pruning of the search space can be done. As all supersets of a closed itemset are known to have a smaller support they are potential pruning candidates. This is especially of interest when reaching the minimum support threshold as all supersets of a closed itemset can be pruned. On the other hand, if an itemset is not closed then there is a least one redundant pair of items. Using the example from above one of the items of *X* is redundant to y. Assuming this redundancy between c and y the itemsets {a,b,c} and {a,b,y} point to the same subset of the data and only one of both has to be investigated.

**Association rule discovery**

In classical ARM, association rules are interesting when they satisfy predefined lower bounds for the support (min_sup) and the confidence (min_conf). However, since a particular alternative measure can be more appropriate for a particular problem, measures other than confidence have been developed to measure the interestingness of a rule [4,5]. In the present study, we used the z-score [6] instead of min_conf. The z-score is an interestingness measure which is an approximation of the exact significance test of the binomial distribution. It is given by

$$z_{AB}=\frac{\mu_{AB}-\mu_{E}}{\sigma_{E}}$$

where $\mu_{AB}=nP(A\cup B)$is the observed frequency that A and B occur jointly;$\mu_{E}=nP(B)P(A)$ is the expected frequency under the assumption of independence between A and B; and $\sigma_{E}^{2}=nP(B)P(A)(1-P(B)P(A))$is the expected variance in the case of independence. Thus, the z-score measures the number of standard deviations $\sigma_{E}$ by which the observed frequency $\mu_{AB}$ deviates from the expected value$\mu_{E}$. In contrast to exact calculation of binomial probability, calculation of the distribution function for each rule is not required. Thus, the measure combines the two crucial features for large-scale computations, i.e. accuracy and efficiency. Furthermore, this measure is of particular interest with respect to genetic data, since statistical approaches are used to account for false positive results in the context of a multiple testing problem.

**Permutation tests.** RUDI implements two kinds of permutation tests. Both aim to test the hypothesis that the association between a phenotype cluster and a genotype pattern is due to chance. Both can be used in addition to the described discovery-replication framework. Within the discovery step, a specific number of phenotype permutations, referred as type 1, can be generated by shuffling the phenotype. The permutation based association results are used to determine an empirical significance value for the association of the original phenotype with the corresponding genotype pattern. Due to the huge search space it is not possible to permute all association results. Thus, it is not possible to define the correct significance level for the association of a genotype pattern with a particular phenotype and an approximative approach is required. RUDI provides a bootstrapping approach [7] to measure the probability of detecting a better genotype-phenotype association by chance and thus receive a significance level. Therefore, n random genotype patterns are generated and tested for association with a particular phenotype. The corresponding association values for this phenotype are compared to the initial association. This permutation method is further referred to as type 2.

**Parameter selection.** Parameter settings for a discovery run are arbitrary. Nonetheless, choosing the parameter settings should follow some practical considerations. First, the discovery run should output only very strong correlations. As the z-score is measured in standard deviations, Chebyshev’s inequality [8] can be used to estimate a useful minimum threshold. Setting the z-score to 5 we expect the algorithm to drop at least 95% of the ‘weaker’ associations. Second, in order to allow for user friendliness and statistically valid results, the support of a single association rule should be sufficiently large. We decided to set the lower bound to 5%. Hence, only genotype patterns that occur in at least 50 patients (5% of the 1,000 patients of the discovery dataset) are considered. Finally, the algorithm should cover as many genotype patterns as possible. The constraining parameter here is the length of the pattern which has the main impact on the runtime. For the given data and parameter selection, we set the value to 3 with respect to the expected runtime (Supplementary Methods, Runtime).

**FURTHER RESULTS**

20,882 candidate rules were found performing the discovery step. Each of these rules reached the user defined threshold for significance (z-score ≥ 5.0) and consists of a multilocus genotype pattern and a set of phenotypes. These sets are further called phenotype clusters, even if the cluster contains one phenotype only, to separate them from the initial phenotype variables. 15 unique phenotype clusters associated with at least one genotype pattern were observed (Table S3). The clusters are disjunct in the sense that a genotype pattern can have only an association with a single phenotype cluster. In contrast, a phenotype cluster can show associations with multiple genotype patterns. The frequencies of the observed patterns range from n=1 for {‘agoraphobia’,’panic disorder’} and {‘agoraphobia’,’social phobia’} to n=9,929 for ‘eating disorder’ (Table S3). Thus, association rules as extracted by ARM cannot be expected to be independent. We further observed significantly more associations per cluster when the phenotype frequency decreased (linear model, p-value=9.126e-03). Thus low frequent phenotypes are more likely to produce false positives. This also illustrates the importance of the replication step for the ARM approach.

Our top findings 3, 4, and 5 (Table 1) are not significant after correction for multiple testing using Bonferroni’s method, but are so when FDR correction is applied. Like our top findings 1 and 2, findings 3, 4, and 5 show statistically significant associations in the combined case-control analysis (Table S5).

Rule #12681 (top3) shows an association with agoraphobia, an anxiety disorder that is characterized by anxiety about being in places or situations from which escape might be difficult, embarrassing, or force panic-like symptoms. The frequencies of the genotype pattern were 7.0% in cases and 7.6% in controls. We observed a statistical difference between the subgroups of patients in the combined analysis of cases (p-value=1.852e-12, OR=3.154 [.95 CI: 2.242-4.384]) that did not remain significant after correction for multiple testing (adjusted p-value=1.747e-01). Conducting case-control analyses for this rule, we observed (a) no association between all BD cases and controls; (b) a significant difference (p-value=0.009, OR=0.747) between the BD_nonAP cases and controls; and (c) a significant difference (p-value=8.637e-08, OR=2.356) when BD_AP cases were compared with controls (Table S5). Similar to our two top findings, we again observed a higher vulnerability in cases where the genotype pattern is present. But for this rule we also observed that the absence of the genotype pattern is slightly protective for the development of the associated phenotype cluster if cases are compared to controls. One of the two genes involved, namely BMP6, has been previously associated with impairments on sustained attention in patients with schizophrenia [9]. But no studies reported an association with a mood disorder.

Rule #12981 (top4) is a similar finding to rule #12978. Two of the three SNPs and the phenotype cluster are identical to our top finding, rule #12978. The non-overlapping SNPs, rs3769745 of rule #12978 and rs8061517 of rule#12981, are located on different chromosomes (Table S7) and do not show a correlation (R-squared = 0.000, D' = 0.015) in the discovery dataset. The frequencies of the genotype pattern were 7.9% in cases and 7.3% in controls. We observed a statistical difference between the subgroups of patients in the combined analysis of cases (p-value=7.539e-12**,** OR=3.503 [.95 CI: 2.371-5.075]) that did not remain significant after multiple testing (adjusted p-value=7.432e-01). Conducting case-control analyses for this rule, we observed (a) no association between all BD cases and controls; (b) no significant difference between the BD_nonED cases and controls; and (c) a significant difference (p-value=2.872e-11, OR=3.372) when BD_ED cases were compared to controls (Table S5). SNP rs8061517, which distinguishes this rule from our top rule, is located within an intergenic region downstream (about 154kbp) of the fat mass and obesity associated gene (FTO), a gene that plays an important role in the central nervous systems. Particularly, an influence on eating behavior (e.g. energy intake, satiety, and binge-eating) and a strong correlation with the body mass index have been observed in humans [10,11].

Rule #6225 (top5) is a redundant finding to rule #6221. While the first two SNPs of the pattern are identical to #6221, the third one, namely rs2844657, is located in the same LD region as rs3130781 (R-squared = 0.793, D' = 0.896, and distance = 85 kb in the discovery dataset), the third SNP of rule #6221. Thus, this finding represents a supporting association to rule #6225.

**SOFTWARE**

The developed open-source software toolset RUDI can be downloaded and used with respect to version 3 of the GNU public licence (GPLv3). Users may distribute and individually adapt the source code. More details including an online tutorial are available at the official web site of RUDI at http://www.rudi-genetics.net.

**ILLUSTRATIONS**

For summary statistics and graphics we used Microsoft Powerpoint, GNU R, PLINK, and RUDI. Figure 1 additionally contains graphics from Benjamin Albiach Galan (http://www.123rf.com/photo_4146037_adn-1.html) and Konstantinos Kokkinis (http://www.123rf.com/photo_772182_person-with-shopping-cart.html) purchased at 123RF Limited (www.123rf.com).

**Members of the Bipolar Disorder Genome Study (BiGS) Consortium**

John R. Kelsoe^1,2^, Tiffany A. Greenwood^1^, Caroline M. Nievergelt^1^, Thomas B. Barrett^1^, Rebecca McKinney^1^, Paul D. Shilling^1^, Nicholas J. Schork^3-5^, Erin N. Smith^3,4^, Cinnamon S.Bloss^3,5^, John Nurnberger^6^, Howard J. Edenberg^7,8^, Tatiana Foroud^8^, Daniel L. Koller^6^, Elliot S. Gershon^9^, Chun-Yu Liu^9^, Judith A. Badner^9^, William Scheftner^10^, William B. Lawson^11^, Evaristus A. Nwulia^11^, Maria Hipolito^11^, William Coryell^12^, John Rice^13^, William Byerley^14^, Francis McMahon^15^, David TW Chen^15^, Thomas G. Schulze^15,16^, Wade Berrettini^17^, James B. Potash^18,19^, Peter P. Zandi^18^, Pamela B. Mahon^18^, Melvin McInnis^20^, David Craig^21^, Szabolcs Szelinger^21^

^1^Department of Psychiatry, University of California, San Diego. ^2^Department of Psychiatry, VA San Diego Healthcare System, La Jolla, CA, USA. ^3^Scripps Genomic Medicine, Scripps Translational Science Institute, and ^4^Department of Molecular and Experimental Medicine, The Scripps Research Institute, and ^5^Scripps Health, La Jolla, CA, USA. ^6^Department of Psychiatry, Indiana University School of Medicine, and ^7^Department of Biochemistry and Molecular Biology, Indiana University School of Medicine, and ^8^Department of Medical and Molecular Genetics, Indiana University School of Medicine, Indianapolis, IN, USA. ^9^Department of Psychiatry, University of Chicago, and ^10^Department of Psychiatry, Rush University, Chicago, IL, USA. ^11^Department of Psychiatry, Howard University, Washington, DC, USA. ^12^Department of Psychiatry, University of Iowa, Iowa City, IA, USA. ^13^Division of Biostatistics, Washington University, St Louis, MO, USA. ^14^Department of Psychiatry, University of California, San Francisco, San Francisco, CA, USA. ^15^Human Genetics Branch, National Institute of Mental Health Intramural Research Program, National Institutes of Health, US Department of Health and Human Services, Bethesda, MD, USA. ^16^Section on Psychiatric Genetics, Department of Psychiatry and Psychotherapy, Georg-August-University, Göttingen, Germany. ^17^Department of Psychiatry, University of Pennsylvania, Philadelphia, PA, USA. ^18^Department of Psychiatry, Johns Hopkins School of Medicine, Baltimore, MD, USA. ^19^Department of Psychiatry, University of Iowa, Iowa City, IO, USA. ^20^Department of Psychiatry, University of Michigan, Ann Arbor, MI, USA. ^21^Neurogenomics Division, The Translational Genomics Research Institute, Phoenix, AZ, USA.

**REFERENCES**

1. Slatkin M (2008) Linkage disequilibrium — understanding the evolutionary past and mapping the medical future. Nature Reviews Genetics 9: 477–485. doi:10.1038/nrg2361.
2. Purcell S, Neale B, Todd-Brown K, Thomas L, Ferreira MAR, et al. (2007) PLINK: a tool set for whole-genome association and population-based linkage analyses. Am J Hum Genet 81: 559–575. doi:10.1086/519795.
3. Guedj M, Nuel G, Prum B (2008) A Note on Allelic Tests in Case-Control Association Studies. Annals of Human Genetics 72: 407–409. doi:10.1111/j.1469-1809.2008.00438.x.
4. Tan P-N, Kumar V, Srivastava J (2002) Selecting the right interestingness measure for association patterns. Proceedings of the eighth ACM SIGKDD international conference on Knowledge discovery and data mining. New York, NY, USA: ACM Press. pp. 32–41. doi:10.1145/775047.775053.
5. Brin S, Motwani R, Silverstein C (1997) Beyond market baskets: generalizing association rules to correlations. Proceedings of the 1997 ACM SIGMOD international conference on Management of data. New York, NY, USA: ACM Press. pp. 265–276. doi:10.1145/253260.253327.
6. Hämäläinen W, Nykänen M (2008) Efficient Discovery of Statistically Significant Association Rules. 2008 Eighth IEEE International Conference on Data Mining. Pisa, Italy. pp. 203–212. doi:10.1109/ICDM.2008.144.
7. Efron B, Tibshirani RJ (1994) An introduction to the bootstrap. New York: Chapman & Hall.
8. Bronstein IN, Semendjajew KA (2008) Taschenbuch der Mathematik. Frankfurt am Main:
9. Lin S-H, Liu C-M, Liu Y-L, Shen-Jang Fann C, Hsiao P-C, et al. (2009) Clustering by neurocognition for fine mapping of the schizophrenia susceptibility loci on chromosome 6p. Genes, Brain and Behavior 8: 785–794. doi:10.1111/j.1601-183X.2009.00523.x.
10. Jacobsson JA, Schiöth HB, Fredriksson R (2012) The impact of intronic single nucleotide polymorphisms and ethnic diversity for studies on the obesity gene FTO. Obes Rev. Available: http://www.ncbi.nlm.nih.gov/pubmed/22931202. Accessed 11 September 2012.
11. Fawcett KA, Barroso I (2010) The genetics of obesity: FTO leads the way. Trends Genet 26: 266–274. doi:10.1016/j.tig.2010.02.006.
