# Supplementary material for: Detecting significant genotype–phenotype association rules in bipolar disorder: market research meets complex genetics
Source: Int J Bipolar Disord. 2018 Nov 11;6:24. doi: 10.1186/s40345-018-0132-x (PMC6230336; doi:10.1186/s40345-018-0132-x)
Supplement: Supplementary file 5 — Additional file 5: Table S3. Overview of all distinct phenotype clusters received from the candidate rules of the discovery step. [file 40345_2018_132_MOESM5_ESM.doc]

**Table S3.** Overview of all distinct phenotype clusters received from the candidate rules of the discovery step.

| GroupID | Size | Phenotype cluster | Frequency in the discovery dataset (in %) |
| --- | --- | --- | --- |
| 12 | 8 | auditory hallucinations | 30.9 |
| 10 | 3 | migraine | 28.9 |
| 14 | 5 | visual hallucinations | 27.0 |
| 9 | 81 | panic disorder | 23.5 |
| 7 | 406 | compulsions | 16.1 |
| 0 | 489 | substance abuse | 14.3 |
| 1 | 1,267 | obsessions | 13.7 |
| 4 | 2,790 | agoraphobia | 12.0 |
| 2 | 2,989 | social phobia | 9.8 |
| 13 | 1 | agoraphobia, panic disorder | 9.6 |
| 3 | 2,908 | simple phobia | 9.4 |
| 8 | 2 | obsessions, compulsions | 8.1 |
| 6 | 9,929 | eating disorder | 6.0 |
| 5 | 1 | agoraphobia, social phobia | 3.9 |
| 11 | 3 | agoraphobia, simple phobia | 3.4 |

The table is sorted descending by the frequency of each cluster in the discovery dataset (GAIN). The column ‘Size’ counts the number of candidate rules to whom the cluster belongs. Thus, e.g. migraine which occurs in 28.9% of the patients is part of 3 candidate rules.
