# Supplementary material for: Detecting significant genotype–phenotype association rules in bipolar disorder: market research meets complex genetics
Source: Int J Bipolar Disord. 2018 Nov 11;6:24. doi: 10.1186/s40345-018-0132-x (PMC6230336; doi:10.1186/s40345-018-0132-x)
Supplement: Supplementary file 6 — Additional file 6: Figure S2. QQ-Plot of all chi-squared values of the replications step (n=20,882). Expected quantiles are based on a 1 degree of freedom distribution. Confidence intervals are based on a 5% error rate. The inflation factor was estimated to be 1.140. [file 40345_2018_132_MOESM6_ESM.docx]

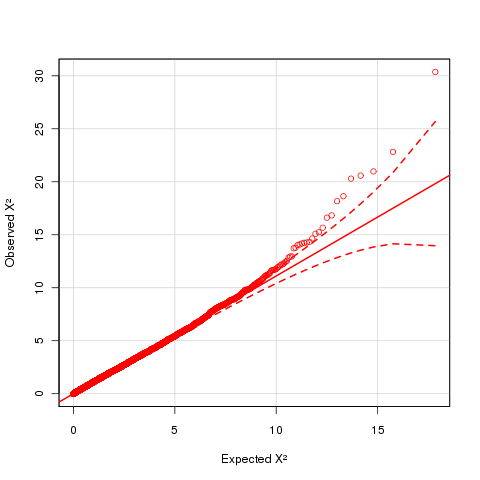


**Figure S2. QQ-Plot of all chi-squared values of the replications step (n=20,882).** Expected quantiles are based on a 1 degree of freedom distribution. Confidence intervals are based on a 5% error rate. The inflation factor was estimated to be 1.140.
