# Supplementary material for: Detecting significant genotype–phenotype association rules in bipolar disorder: market research meets complex genetics
Source: Int J Bipolar Disord. 2018 Nov 11;6:24. doi: 10.1186/s40345-018-0132-x (PMC6230336; doi:10.1186/s40345-018-0132-x)
Supplement: Supplementary file 7 — Additional file 7: Table S4. Association results for each data set of our top finding, pattern #12978. [file 40345_2018_132_MOESM7_ESM.doc]

**Table S4. Association results for each data set of our top finding, pattern #12978.**

| Sample | N | Odds ratio [.95 CI] | sup(G) | sup(P) | p_chisq |
| --- | --- | --- | --- | --- | --- |
| *Discovery* GAIN | 1,000 | 5.648 [2.672-11.229] | 0.052 | 0.060 | 1.006e-07 |
| *Replication*  TGEN+BOMA | 1,835 | 3.566 [2.169-5.681] | 0.071 | 0.072 | 3.576e-08 |
| TGEN | 1,190 | 3.276 [1.902-5.467] | 0.074 | 0.104 | 3.288e-06 |
| BoMa | 645 | 9.297 [1.740-40.953] | 0.065 | 0.012 | 3.507e-04* |
| Controls | 2,744 | - | 0.055 | - | - |
| GAIN | 1,033 | - | 0.052 | - | - |
| TGEN | 401 | - | 0.052 | - | - |
| BOMA | 1,310 | - | 0.058 | - | - |

sup(G) is the support/frequency of the genotype pattern and sup(P) the support/frequency of the phenotype cluster. p_chisq is the p-value calculated on the basis of a 2x2 contingency table. Controls were merged from all 3 control samples (Supplementary Table 1). *Chi-squared approximation may be incorrect due to low cell counts.
