# Supplementary material for: Detecting significant genotype–phenotype association rules in bipolar disorder: market research meets complex genetics
Source: Int J Bipolar Disord. 2018 Nov 11;6:24. doi: 10.1186/s40345-018-0132-x (PMC6230336; doi:10.1186/s40345-018-0132-x)
Supplement: Supplementary file 8 — Additional file 8: Table S7. Details regarding the genotype patterns of the top 5 association rules. [file 40345_2018_132_MOESM8_ESM.doc]

**Table S7.** Details regarding the genotype patterns of the top 5 association rules.

| PID | Genotype | Source [I5/A6] | Chr | Gene | Gene name | AF (dbSNP) | Function class |
| --- | --- | --- | --- | --- | --- | --- | --- |
| #12978 | rs6733011_A_0 | Imputed / Imputed | 2q11.2 | KIAA1211L | KIAA1211-like | A=0.438/958 | intron-variant |
|  | rs4113925_T_0 | Genotyped / Imputed | 12q24.1 | TBX5 | **T-box 5** | T=0.395/864 | intron-variant |
|  | rs3769745_T_0 | Genotyped / Genotyped | 2q11.2 | CNGA3 | **cyclic nucleotide gated channel alpha 3** | T=0.241/527 | intron-variant |
| #6221 | rs858057_G_0 | Imputed / Imputed | 20p11.21 | - | - | A=0.410/897 | intergenic |
|  | rs4757144_G_0 | Imputed / Imputed | 11p15 | ARNTL | **aryl hydrocarbon receptor nuclear translocator-like** | A=0.410/896 | intron-variant |
|  | rs3130781_C_0 | Imputed / Genotyped | 6p21.33 | DPCR1 | **diffuse panbronchiolitis critical region 1** | C=0.181/226 | intron-variant |
| #12681 | rs1083672_G_0 | Imputed / Imputed | 3q28 | - | - | G=0.308/674 | intergenic |
|  | rs6539349_G_12 | Genotyped / Genotyped | 12q23.3 | BTBD11 | BTB (POZ) domain containing 11 | G=0.353/772 | intron-variant |
|  | rs4960348_C_12 | Imputed / Imputed | 6p24.3 | BMP6 | bone morphogenetic protein 6 | C=0.208/456 | intron-variant |
| #12981 | rs6733011_A_0 | Imputed / Imputed | 2q11.2 | KIAA1211L | KIAA1211-like | A=0.438/958 | intron-variant |
|  | rs4113925_T_0 | Genotyped / Imputed | 12q24.1 | TBX5 | **T-box 5** | T=0.395/864 | intron-variant |
|  | rs8061517_G_0 | Genotyped / Genotyped | 16q12.2 | - | - | G=0.254/554 | intergenic |
| #6225 | rs858057_G_0 | Imputed / Imputed | 20p11.21 | - | - | A=0.410/897 | intergenic |
|  | rs4757144_G_0 | Imputed / Imputed | 11p15 | ARNTL | aryl hydrocarbon receptor nuclear translocator-like | A=0.410/896 | intron-variant |
|  | rs2844657_G_0 | Genotyped / Imputed | 6p21.3 | - | - | G=0.154/336 | intergenic |

SNP to Gene annotation via dbSNP. Abbreviations: Chr = chromosomal region; AF = allele frequency; I5 = Illumina HumanHap550; A6 = Affymetrix 6.0
