# Supplementary material for: Detecting significant genotype–phenotype association rules in bipolar disorder: market research meets complex genetics
Source: Int J Bipolar Disord. 2018 Nov 11;6:24. doi: 10.1186/s40345-018-0132-x (PMC6230336; doi:10.1186/s40345-018-0132-x)
Supplement: Supplementary file 9 — Additional file 9: Table S6. Single SNP association results for each SNP of our two test-wide significant findings (Bonferroni) regarding the phenotype cluster of the corresponding association rule. [file 40345_2018_132_MOESM9_ESM.doc]

**Table S6. Single SNP association results for each SNP of our two test-wide significant findings (Bonferroni) regarding the phenotype cluster of the corresponding association rule.**

| PID | Sample | SNP | A1/A2 | AFF | UNAFF | p-value | odds ratio [.95 CI] |
| --- | --- | --- | --- | --- | --- | --- | --- |
| #12978 | GAIN | rs6733011 | A/G | 47/73 | 849/1025 | 0.1871 | 0.778 [0.530-1.132] |
|  |  | rs4113925 | T/C | 29/91 | 737/1137 | 7.964e-04 | 0.494 [0.316-0.748] |
|  |  | rs3769745 | T/C | 27/93 | 451/1423 | 0.702 | 0.920 [0.581-1.411] |
|  | TGEN | rs6733011 | A/G | 106/142 | 985/1147 | 0.3104 | 0.870 [0.665-1.133] |
|  |  | rs4113925 | T/C | 91/157 | 888/1244 | 0.1383 | 0.813 [0.617-1.065] |
|  |  | rs3769745 | T/C | 50/198 | 460/1672 | 0.6012 | 0.920 [0.657-1.266] |
|  | BoMa | rs6733011 | A/G | 5/11 | 551/723 | 0.3342 | 0.607 [0.186-1.703] |
|  |  | rs4113925 | T/C | 7/9 | 500/774 | 0.7098 | 1.210 [0.422-3.317] |
|  |  | rs3769745 | T/C | 2/14 | 293/981 | 0.3272* | 0.510 [0.073-1.859] |
|  |  |  |  |  |  |  |  |
| #6221 | GAIN | rs858057 | G/A | 84/100 | 901/909 | 0.2944 | 0.848 [0.624-1.149] |
|  |  | rs4757144 | G/A | 62/122 | 719/1091 | 0.1037 | 0.772 [0.558-1.059] |
|  |  | rs3130781 | C/T | 36/148 | 348/1462 | 0.9133 | 1.025 [0.690-1.487] |
|  | TGEN | rs858057 | G/A | 154/206 | 980/1040 | 0.04245 | 0.794 [0.632-0.995] |
|  |  | rs4757144 | G/A | 143/217 | 825/1195 | 0.6808 | 0.955 [0.758-1.200] |
|  |  | rs3130781 | C/T | 59/301 | 400/1620 | 0.124 | 0.795 [0.585-1.066] |
|  | BoMa | rs858057 | G/A | 4/12 | 618/656 | 0.07042 | 0.363 [0.098-1.064] |
|  |  | rs4757144 | G/A | 6/10 | 478/796 | 0.9986 | 1.010 [0.335-2.776] |
|  |  | rs3130781 | C/T | 3/13 | 275/999 | 0.7905* | 0.872 [0.190-2.765] |

The results (p-values) are based on the Cochran-Armitage trend test and have been performed using PLINK^1^. Abbreviations: A1 = reference allele; A2 = corresponding allele; AFF = Affected group; UNAFF = Unaffected group. *Chi-squared approximation may be incorrect due to low cell count.
