# Supplementary material for: Detecting significant genotype–phenotype association rules in bipolar disorder: market research meets complex genetics
Source: Int J Bipolar Disord. 2018 Nov 11;6:24. doi: 10.1186/s40345-018-0132-x (PMC6230336; doi:10.1186/s40345-018-0132-x)
Supplement: Supplementary file 10 — Additional file 10: Table S5. Association results of the case-control analyses for the top 5 replication patterns. [file 40345_2018_132_MOESM10_ESM.doc]

**Table S5. Association results of the case-control analyses for the top 5 replication patterns.**

| PID | Test | n_cases  (G/g) | n_controls  (G/g) | p-value | odds ratio [.95 CI] |
| --- | --- | --- | --- | --- | --- |
| #12978 | BD_ED vs. controls | 37/155 | 151/2593 | 4.937e-14 | 4.107 [2.735-6.040] |
|  | BD_nonED vs. controls | 145/2498 | 151/2593 | 0.979 | 0.997 [0.788-1.260] |
|  | BD vs. controls | 182/2653 | 151/2593 | 0.148 | 1.178 [0.943-1.473] |
|  |  |  |  |  |  |
| #6221 | BD_SP vs. controls | 46/236 | 158/2586 | 1.686e-11 | 3.195 [2.220-4.523] |
|  | BD_nonSP vs. controls | 133/2420 | 158/2586 | 0.381 | 0.900 [0.709-1.140] |
|  | BD vs. controls | 179/2656 | 158/2586 | 0.384 | 1.103 [0.885-1.376] |
|  |  |  |  |  |  |
| #12681 | BD_AP vs. controls | 55/285 | 208/2536 | 8.637e-08 | 2.356 [1.695-3.230] |
|  | BD_nonAP vs. controls | 144/2351 | 208/2536 | 0.009 | 0.747 [0.599-0.930] |
|  | BD vs. controls | 199/2636 | 208/2536 | 0.421 | 0.920 [0.752-1.127] |
|  |  |  |  |  |  |
| #12981 | BD_ED vs. controls | 40/152 | 199/2545 | 2.872e-11 | 3.372 [2.287-4.876] |
|  | BD_nonED vs. controls | 185/2458 | 199/2545 | 0.719 | 0.963 [0.782-1.185] |
|  | BD vs. controls | 225/2610 | 199/2545 | 0.335 | 1.102 [0.904-1.345] |
|  |  |  |  |  |  |
| #6225 | BD_SP vs. controls | 46/236 | 164/2580 | 7.844e-11 | 3.071 [2.137-4.342] |
|  | BD_nonSP vs. controls | 135/2418 | 164/2580 | 0.278 | 0.879 [0.694-1.110] |
|  | BD vs. controls | 181/2654 | 164/2580 | 0.527 | 1.073 [0.863-1.335] |

The statistics are based on chi-squared tests on contingency tables. The analyses have been performed using all patients and all controls across the three samples: GAIN, TGEN, and BoMa. Abbreviations: G = genotype pattern present; g = genotype pattern not present; BD = bipolar disorder; ED = ‘eating disorder’; SP = ‘simple phobia’; AP = ‘agoraphobia’; CI = confidence interval; BD_ED represents the supgroup of BD patients that have a comorbid eating disorder, while BD_nonED represents the complementary subgroup without ED. This applies accordingly to all other supgroups.
